# Data-Efficient Computational Pathology Platform for Faster and Cheaper Breast Cancer Subtype Identifications: Development of a Deep Learning Model

**DOI:** 10.2196/45547

**Published:** 2023-09-05

**Authors:** Kideog Bae, Young Seok Jeon, Yul Hwangbo, Chong Woo Yoo, Nayoung Han, Mengling Feng

**Affiliations:** 1 Healthcare AI Team Healthcare Platform Center National Cancer Center Goyang-si, Gyeonggi-do Republic of Korea; 2 Institute of Data Science National University of Singapore Singapore Singapore; 3 Department of Cancer AI & Digital Health Graduate School of Cancer Science and Policy National Cancer Center Goyang-si, Gyeonggi-do Republic of Korea; 4 Department of Pathology National Cancer Center Hospital National Cancer Center Goyang-si, Gyeonggi-do Republic of Korea; 5 Saw Swee Hock School of Public Health National University of Singapore Singapore Singapore

**Keywords:** deep learning, self-supervised learning, immunohistochemical staining, machine learning, histology, pathology, computation, predict, diagnosis, diagnose, carcinoma, cancer, oncology, breast cancer

## Abstract

**Background:**

Breast cancer subtyping is a crucial step in determining therapeutic options, but the molecular examination based on immunohistochemical staining is expensive and time-consuming. Deep learning opens up the possibility to predict the subtypes based on the morphological information from hematoxylin and eosin staining, a much cheaper and faster alternative. However, training the predictive model conventionally requires a large number of histology images, which is challenging to collect by a single institute.

**Objective:**

We aimed to develop a data-efficient computational pathology platform, 3DHistoNet, which is capable of learning from z-stacked histology images to accurately predict breast cancer subtypes with a small sample size.

**Methods:**

We retrospectively examined 401 cases of patients with primary breast carcinoma diagnosed between 2018 and 2020 at the Department of Pathology, National Cancer Center, South Korea. Pathology slides of the patients with breast carcinoma were prepared according to the standard protocols. Age, gender, histologic grade, hormone receptor (estrogen receptor [ER], progesterone receptor [PR], and androgen receptor [AR]) status, erb-B2 receptor tyrosine kinase 2 (HER2) status, and Ki-67 index were evaluated by reviewing medical charts and pathological records.

**Results:**

The area under the receiver operating characteristic curve and decision curve were analyzed to evaluate the performance of our 3DHistoNet platform for predicting the ER, PR, AR, HER2, and Ki67 subtype biomarkers with 5-fold cross-validation. We demonstrated that 3DHistoNet can predict all clinically important biomarkers (ER, PR, AR, HER2, and Ki67) with performance exceeding the conventional multiple instance learning models by a considerable margin (area under the receiver operating characteristic curve: 0.75-0.91 vs 0.67-0.8). We further showed that our z-stack histology scanning method can make up for insufficient training data sets without any additional cost incurred. Finally, 3DHistoNet offered an additional capability to generate attention maps that reveal correlations between Ki67 and histomorphological features, which renders the hematoxylin and eosin image in higher fidelity to the pathologist.

**Conclusions:**

Our stand-alone, data-efficient pathology platform that can both generate z-stacked images and predict key biomarkers is an appealing tool for breast cancer diagnosis. Its development would encourage morphology-based diagnosis, which is faster, cheaper, and less error-prone compared to the protein quantification method based on immunohistochemical staining.

## Introduction

### Rationale

Breast cancer is the fourth most frequent cause of death worldwide [1]. Invasive breast cancer from the heterogeneous group of breast epithelial malignancies shows distinct outcomes and responses to therapy due to the presence of subtypes, which can be defined based on the biomarker expression status [2]. These biomarkers include estrogen receptor (ER), progesterone receptor (PR), androgen receptor (AR), erb-B2 receptor tyrosine kinase 2 (ERBB2 or commonly called HER2), and antigen Ki67. In clinical practice, biomarker expressions in invasive breast cancer can be evaluated using immunohistochemical (IHC) staining. IHC has been a routine clinical process for a long time, but it is still susceptible to the pathologist’s subjectivity and human errors [3]. Besides, due to the high specificity of IHC staining that can only identify a single biomarker at a time, multiple rounds of IHC staining are often required and, thus, deemed to be costly and time-consuming.

Hematoxylin and eosin (H&E) staining is another routine clinical procedure for primary cancer diagnosis (eg, cancer vs benign) and is generally performed prior to IHC staining. Although it has been suspected that H&E-stained slides may reflect the characteristic phenotypes of the prognostic biomarkers [4,5], recent deep learning models show the possibility of capturing latent features from H&E images and achieving reasonably accurate prediction of the subtype biomarkers [6-8], potentially saving clinical resources. However, these models rely on a massive number of training samples that often require data collection from multiple institutes. In practice, this approach is challenging due to a number of reasons: (1) there are inevitable data variations among institutes due to differences in equipment models and protocols adopted by each institute, and (2) data sharing across institutes faces data privacy and security issues. Such a dilemma may be overcome by developing a data-efficient model that is capable of learning from smaller training samples that still maintains high prediction accuracy.

Recent deep learning research has been focusing on the analysis of 3D medical images [9-12]. This is because 3D images offer additional information that may not be unveiled in the 2D images, leading to a more accurate classification of tissues. Likewise, 3D visualization at the cellular level is able to capture the complete morphology of nuclei, which is closely associated with cancer pathology and medical complications [13,14]. However, 3D histology images are less popular, as 3D histomorphological features rarely appear intuitive to human eyes. Hence, 2D histology image-based cancer prediction models have thus far been proposed [6-8]. Nevertheless, we hypothesized that these 3D features carry useful information that can help our proposed deep learning model to learn more effectively, even from a small set of data.

### Objectives

In this study, we developed a data-efficient computational pathology platform, 3DHistoNet, to identify all 5 biomarkers (ER, PR, AR, HER2, and Ki67) associated with breast cancer subtypes. We aimed to demonstrate that our model can (1) generate z-stacked histology images suitable as a 3D data set for the training of our model; (2) harness a 3D data set to achieve improved prediction performance even with a smaller sample size; and (3) additionally produce attention maps that visualize the morphological characteristics of various prognostic biomarkers, thereby allowing pathologists to directly gain molecular information from H&E slides alone.

## Methods

**Figure 1:**
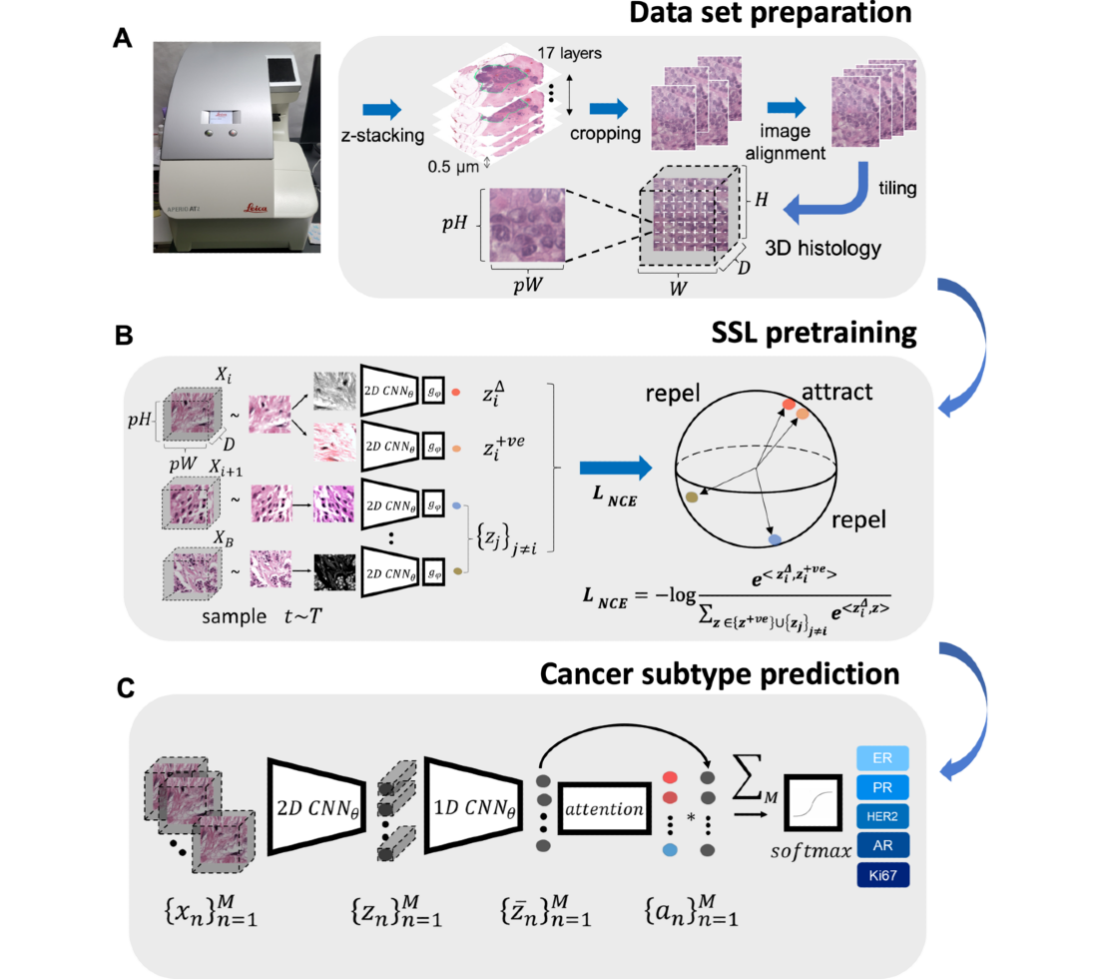
Schematics of 3DHistoNet for the prediction of prognostic biomarkers from hematoxylin and eosin slides. The model consists of 3 stages: (A) the preparation of z-stacked whole slide tissue images, (B) self-supervised feature extraction from z-stacked tissue images, and (C) attention-based prediction model. AR: androgen receptor; CNN: convolutional neural network; ER: estrogen receptor; HER2: erb-B2 receptor tyrosine kinase 2; PR: progesterone receptor; SSL: self-supervised learning.

As shown in Figure 1, our pathology platform is composed of 3 stages: the preparation of the z-stacked whole slide tissue image data set (Figure 1A), self-supervised feature extraction from z-stacked tissue images (Figure 1B), and an attention-based prediction model (Figure 1C). The following subsections describe each of the stages. This study is reported according to the Guidelines for Developing and Reporting Machine Learning Predictive Models in Biomedical Research [15].

### Data Source

We retrospectively examined 401 cases of patients with primary breast carcinoma diagnosed between 2018 and 2020 at the Department of Pathology, National Cancer Center, South Korea. Pathological diagnoses of the specimens were performed by a breast pathologist following the World Health Organization guidelines and the American Joint Committee on Cancer staging manual (8th edition). Glass slides; medical charts; and pathological records including histologic grade, hormone receptor (ER, PR, and AR) status, HER2 status, and Ki-67 index were reviewed by another pathologist before collecting cases. Patients who meet any of the following conditions were excluded: (1) whole slide images were not available, (2) malignant lesions were not found, and (3) diagnosed as having breast cancer. Positive ratios for each biomarker were as follows: ER (313/401, 78.1%), PR (279/401, 69.6%), AR (353/401, 88%), HER2 (305/401, 76%), and Ki67 (258/401, 64.3%).

### Ethics Approval

The retrospective study protocol was approved by the Institutional Review Board of the National Cancer Center (NCC2021-0283).

### Preparation of z-Stacked Whole Slide Images for Model Building

We scanned the entire morphology of the H&E-stained tissue specimens using a pathology slide scanner (Aperio AT2, Leica Biosystems) set at a magnification of 40× (pixel size of 0.25 µm). At each focal plane, the lateral (x-y) dimensions were scanned. After completion, the focal plane was shifted by moving the objective lens axially at an interval of 0.5 µm to stack the whole slide scanning. To cover the entire depth of focus determined by the tissue thickness (3-4 µm) and further extended by the axial resolution (~2 µm) of the objective lens, 17 z-stack layers (~8 µm) were obtained. Due to the insufficient precision of the translational stage in the scanner, misalignment along the stack layer may occur. As a correction, we used an image registration algorithm with affine transformation provided by ImageJ. As breast cancer subtypes are a subset of cancer, we confined our region of interest to the cancer region as annotated by trained, certified pathologists. The region was then cropped into 256×256 image tiles without any overlap for a multiple instance learning (MIL) approach. The total number of tiles obtained was 187,921 from 401 specimens.

### Predictive Models

We used a self-supervised learning (SSL) approach to train a neural network that extracts low-dimensional features from z-stacked H&E image stacks in a label-free way. Specifically, we adopted the recently proposed Simple Framework for Contrastive Learning of Visual Representations (SimCLR) [16] as our SSL framework. SimCLR learns to extract abstract features from H&E scans by maximizing the “agreement” between the altered views of the same input and minimizing it otherwise.

Figure 1B illustrates the application of SimCLR to our task of extracting features from z-stacked H&E tiles. First, we generated altered views for each z-stacked H&E tile by applying a set of mild image transformations, such as affine transform, color jittering, resizing, and cropping, which still preserved most of the key semantics of the original input tile. These altered views were then passed through a neural network, referred to as 2D convolutional neural network (CNN) in the figure, which outputs low-dimensional features for each of the views, shown as colored dots in the figure. We used InfoNCE loss [17] as the training objective that “attracts” the features generated from the same input tile and “repels” the features from different input tile sources. This “attract” and “repel” process is visualized in the figure as the proximity of colored points within a sphere. A more formal explanation that accompanies mathematical notations and definitions is available in Multimedia Appendix 1 [16-19].

After training the feature extractor neural network 2D CNN as described in the previous subsection, the next step was to use the extracted features to train another set of neural networks for the actual cancer subtypes prediction task. The overview is shown in Figure 1C. First, we extracted features from z-stacked input tiles using the SSL pretrained neural network 2D CNN. The extracted features were passed through a set of prediction modules comprising 3 submodules: (1) a 1D CNN module that integrates z-stacked representations into a single representation; (2) an attention module that generates a heatmap, which assigns a higher value to the representations that contribute strongly to prediction; and (3) a classifier layer that produces a probability of different cancer subtypes.

Regarding the z-stacked representations as multichannel 1D signals that may contain informative interactions across the signals, we applied 1D CNN to find such interactions across the stacks and integrated them into a single representation, represented as gray dots in the figure. The 1D CNN is comprised of 2 CNN blocks. Each block contains a 1D convolutional layer and a rectified linear unit layer. Taking the set of integrated representations as inputs, an attention module generates scores that measure the relative importance of each representation to the final prediction. The attention module not only helps to accelerate the model training but also assists health care practitioners in identifying potential areas that may require further focus.

Finally, the computed attention scores were used to perform a weighted average across the representations set and subsequently fed into a classifier module comprised of a fully connected layer and a softmax layer to produce cancer subtype probabilities. All 3 submodules were trained based on latent features of the H&E image stack in an end-to-end fashion with a cross-entropy loss function that matches the prediction with the ground-truth cancer subtype labels (ER, PR, AR, HER2, and Ki67). A more formal explanation that accompanies mathematical notations and definitions is available in Multimedia Appendix 1.

### Model Training Setting

We used ResNet50 as our feature extractor neural network [18]. During SSL pretraining, we set the tile size to 256,set the training batch size to 256, and trained for 250 epochs. For generating different views from a source tile, we applied random cropping with a scale between a factor of 0.4 to 1; rotations of 0°, 90°, 180°, and 270°; horizontal flipping; color jittering; RGB to grayscale; gaussian blur; and solarization. Additionally, to accelerate the training speed and reduce large memory consumption, we used mixed-precision training, which combines single precision (32 bit) and half precision (16 bit). We optimize the model using the Adam optimizer [20] with a learning rate of 0.0003.

In the cancer subtype prediction task, we trained the 3 modules of our model with a batch size of 1 because each specimen has a varying tile number depending on the specimen size. No augmentation is applied to the features extracted from the tiles as the features are no longer humanly interpretable, making it difficult to know which augmentation preserves the key contents of the representation. We oversampled minority labels to address the class imbalance issue. We optimized the model using Adam optimizer [20] with a learning rate of 0.0001. We measured the variability of the model prediction with 5-fold cross-validation as internal validation. We also implemented an identical 5-fold split during the SSL pretraining step to ensure that there is no bias favoring our proposed approach over other baseline approaches. The average receiver operating characteristic curve and its area under the curve (AUC) over the 5-fold validation results were used to measure the model performance. Our 3DHistoNet was implemented in PyTorch [21] (version >1.9.0) and trained on a single NVIDIA Tesla V100 GPU with 32GB memory.

For comparison with our model, we used ImageNet-pretrained ResNet50 (IMAGENET), which is officially available in PyTorch as the baseline feature extractor. During the IMAGENET pretraining, a standard set of data augmentation techniques (random crop, resize, rotation, and intensity adjustments) were applied. For model optimization, stochastic gradient descent was applied with a learning rate of 0.1, momentum of 0.9, and decay rate of 0.99998. A minibatch size of 256 was used during pretraining.

## Results

### Evaluation of the Model Performance of 3DHistoNet

**Figure 2:**
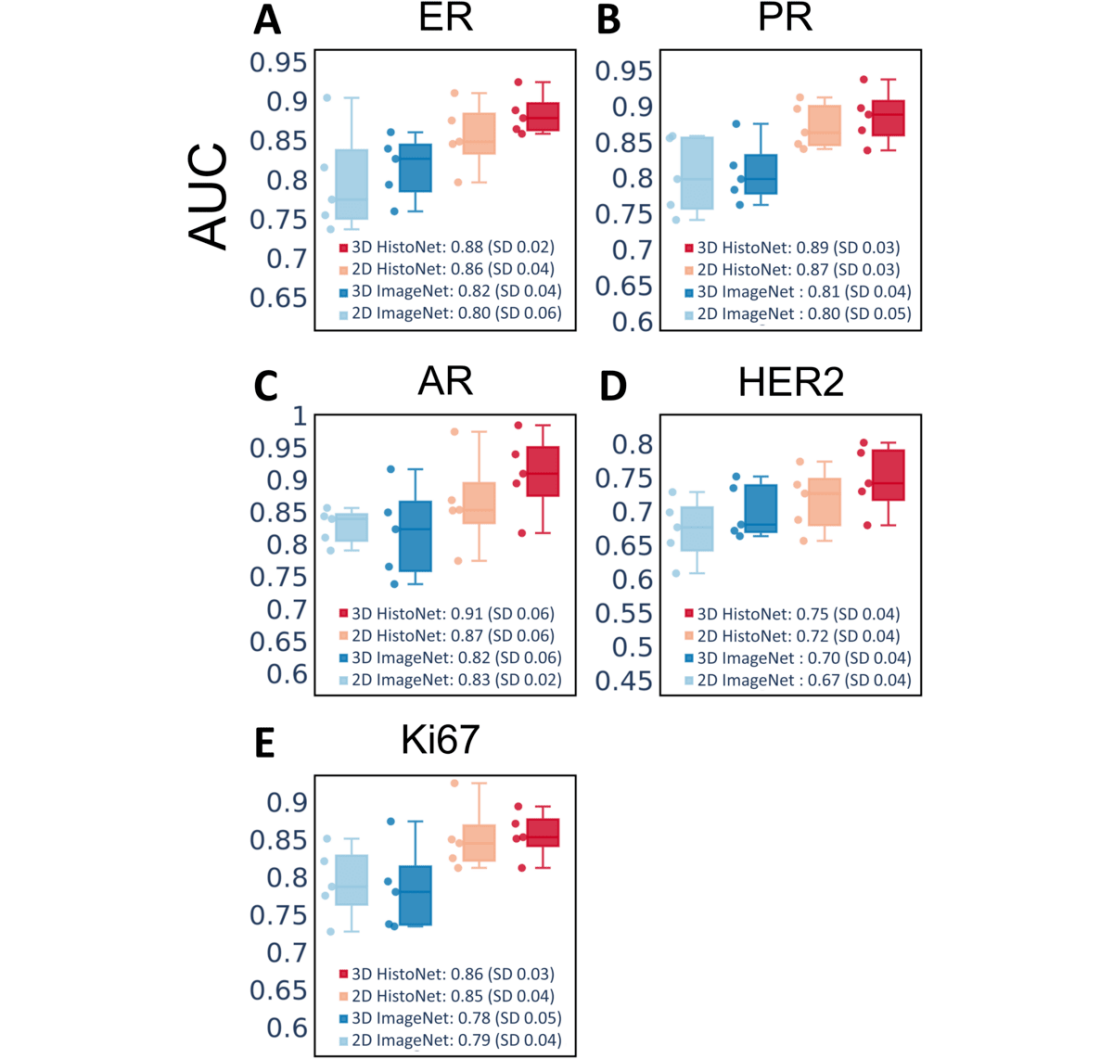
3DHistoNet shows superior performance in the prediction of prognostic biomarkers (ER, PR, AR, HER2, and Ki67) in comparison with the conventional supervised learning model. Box plots of the area under the curve (AUC) are plotted to compare the performance of 3DHistoNet with ImageNet-pretrained ResNet50 model (IMAGENET) when trained with 2D and 3D histology data sets, respectively (n=401). AR: androgen receptor; ER: estrogen receptor; HER2: erb-B2 receptor tyrosine kinase 2; PR: progesterone receptor.

The classification capability of 3DHistoNet for all prognostic biomarkers, including ER, PR, AR, HER2, and Ki67, is shown in Figure 2. The AUC ranged from 0.75 to 0.91, demonstrating outstanding prediction accuracy despite being trained with a small data set (n=401). For comparison, we repeated the experiments with a single best-focused image from the image stack as representative of a 2D data set or with an IMAGENET model in place of SimCLR. We found that 3DHistoNet significantly outperformed IMAGENET regardless of the data type and target class, suggesting the superiority of the SSL model over the conventional supervised learning model. Our results also showed that the use of the image stack generally enhanced the classification performance compared to the 2D counterparts. On the other hand, both 3DHistoNet and IMAGENET scored an ascending order of AR, PR, ER, Ki67, and HER2 in terms of prediction performance, implying that the difficulty of the classification task is dependent on the characteristic features associated with the biomarkers.

**Figure 3:**
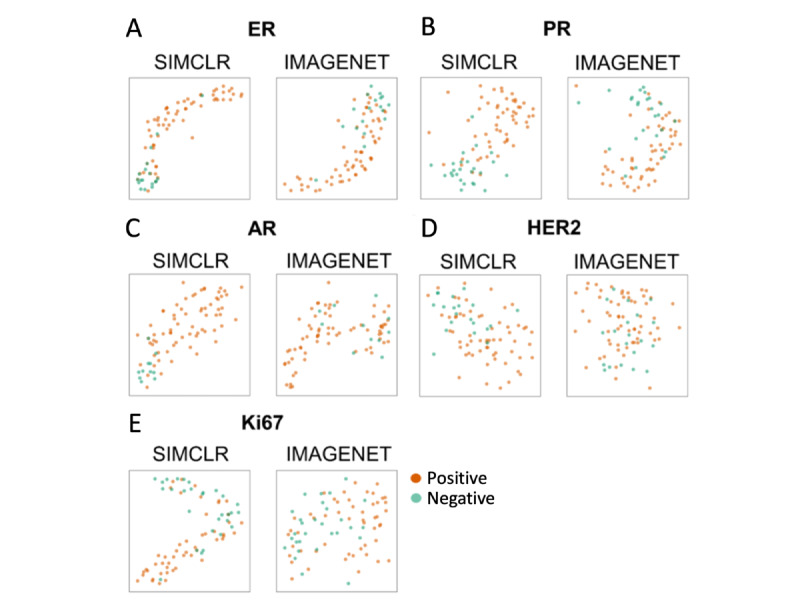
t-Distributed Stochastic Neighbor Embedding (tSNE) analysis on low-dimensional features of the 3D data set from feature extraction modules of 3DHistoNet (SimCLR) and IMAGENET. 3DHistoNet attained higher discrimination power at the feature extraction stage. AR: androgen receptor; ER: estrogen receptor; HER2: erb-B2 receptor tyrosine kinase 2; IMAGENET: ImageNet-pretrained ResNet50; PR: progesterone receptor; SimCLR: Simple Framework for Contrastive Learning of Visual Representations.

We further performed a t-Distributed Stochastic Neighbor Embedding analysis (Figure 3) to compare the discrimination power of the 2 models without the confounding effects from the downstream layers (ie, prediction model). The results showed that 3DHistoNet forms more distinguishable clusters compared to IMAGENET, confirming the higher discrimination capability of the former. This trend is consistent with Figure 2, whereby AR showed the most contrasting clusters (Figure 3C) and HER2 showed the most overlapping features (Figure 3D).

It has been shown that the MIL approach is suitable for the classification of histology images [22,23]. The aggregation of the prediction results of patch images can effectively diagnose the whole slide image that these patches belong to. With a few choices of MIL algorithms (MeanPool, MaxPool, and Attention) given [24], we investigated the optimal algorithm for our classification task (Table 1). In general, the attention algorithm scored the highest accuracy for AR, PR, and HER2; although overall, no statistical significance in the performance of the 3 algorithms was observed. The result could imply that the high classification performance is mainly attributed to our SSL module. However, further validation with a sufficiently large data set remains to be done in the future. For our implementation, we chose the attention algorithm owning to its additional visualization function to highlight key diagnostic features.

**Table 1:** Comparison of the 3DHistoNet performance with different multiple instance learning algorithms (MeanPool, MaxPool, and Attention).

Biomarker	MeanPool, mean (SD)	MaxPool, mean (SD)	Attention, mean (SD)
ER^a^	0.882 (0.041)	*0.89 (0.044)* ^b^	0.883 (0.026)
PR^c^	0.882 (0.04)	0.875 (0.032)	*0.888 (0.036)*
AR^d^	0.879 (0.093)	0.893 (0.082)	*0.906 (0.075)*
HER2^e^	0.732 (0.054)	0.739 (0.053)	*0.748 (0.049)*
Ki67	*0.861 (0.033)*	0.823 (0.012)	0.857 (0.03)

^a^ER: estrogen receptor.

^b^Italicization represents the multiple instance learning algorithm with the highest performance for each biomarker.

^c^PR: progesterone receptor.

^d^AR: androgen receptor.

^e^HER2: erb-B2 receptor tyrosine kinase 2.

### Assessment of the Data Efficiency of 3DHistoNet

**Figure 4:**
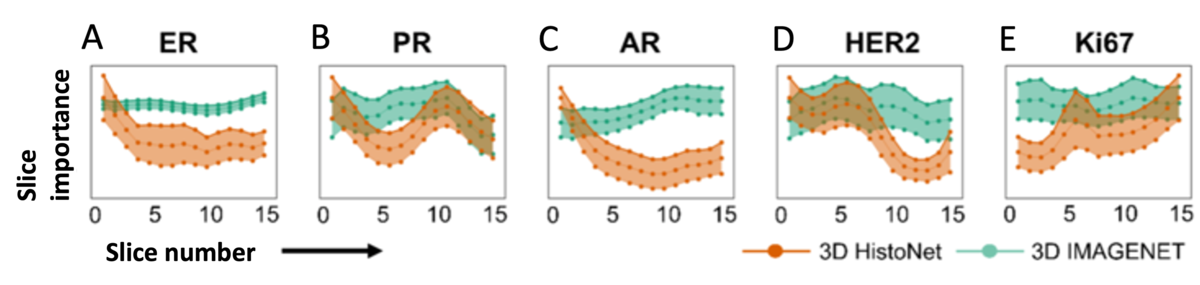
A graph that indicates the slice-wise feature importance in predicting biomarkers, which is computed by aggregating the Gradient-weighted Class Activation Mapping (Grad-CAM) score across all spatial axes (height and width) and tiles (A-E). The shaded region indicates the empirical SD of the slice-wise feature importance estimated using a held-out test set. AR: androgen receptor; ER: estrogen receptor; HER2: erb-B2 receptor tyrosine kinase 2; IMAGENET: ImageNet-pretrained ResNet50; PR: progesterone receptor.

The results in Figure 2 imply that the benefit of using the image stack is greater with 3DHistoNet than with IMAGENET, suggesting that 3DHistoNet is more efficient in the extraction of relevant information from the image stack. To prove this, we evaluated the average contributions of z-slices to the biomarker prediction (Figure 4). The result shows that 3DHistoNet referred to more significant slices at different levels, whereas IMAGENET assigned equal importance to all layers, implying that it cannot extract significant information from the multiple layers. Further, we also noticed that 3DHistoNet showed different referencing patterns depending on the biomarkers, substantiating its capability to predict different biomarkers with the same architecture.

**Figure 5:**
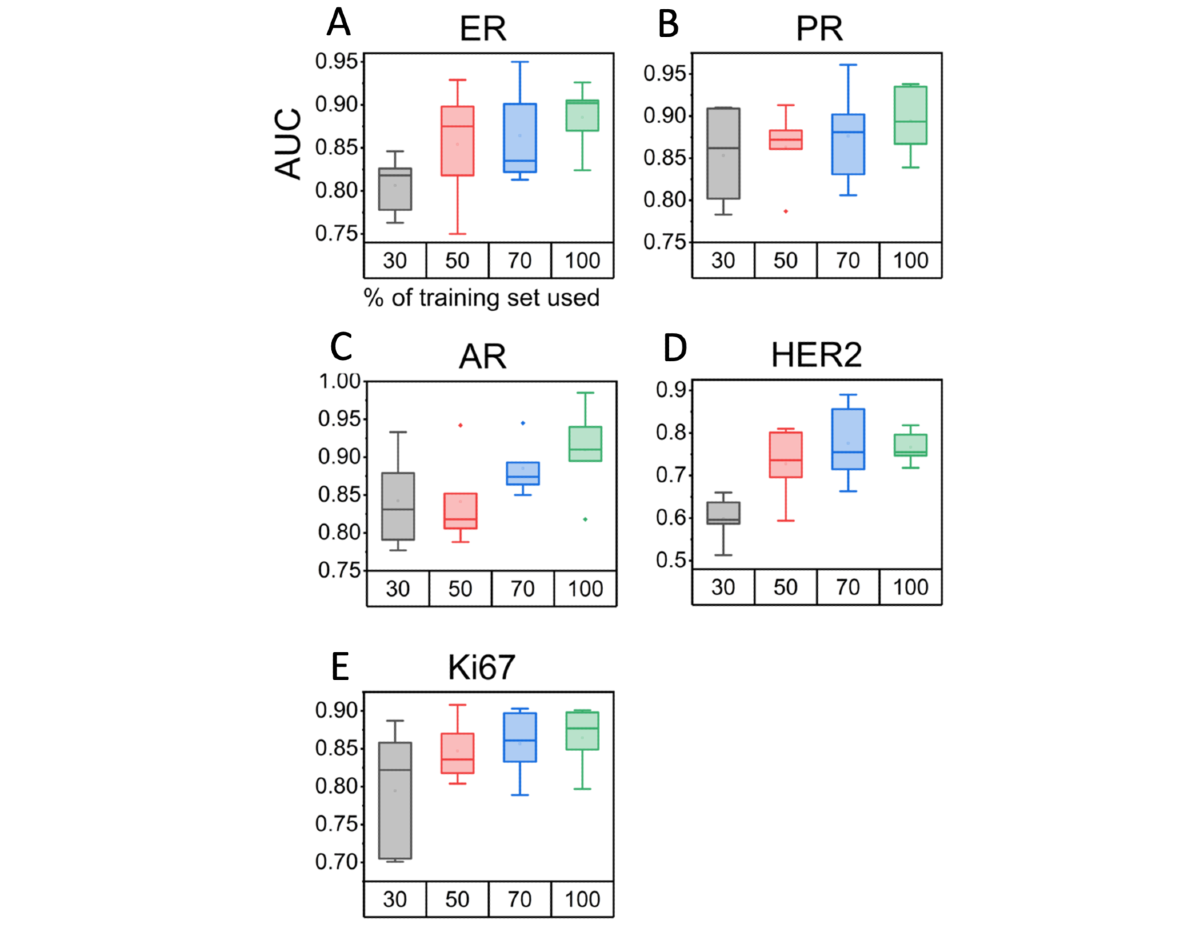
Box plots based on the area under the curve (AUC) to show the data set size–dependent performance of 3DHistoNet in terms of the 5-fold average AUCs. The model was independently trained with 30% (n=120 cases), 50% (n=200 cases), 70% (n=280 cases), and 100% (n=401 cases) of the 3D histology data set. AR: androgen receptor; ER: estrogen receptor; HER2: erb-B2 receptor tyrosine kinase 2; PR: progesterone receptor.

We also proved that the z-stacked histology image helped overcome the shortage of training data set, which is encountered as a common limitation imposed on model performance. We sequentially sampled subsets of the training data set in the proportion of 30%, 50%, and 70% of the total number of cases in the training set (Figure 5). The performance of 3DHistoNet generally reached the optimal level with 70% of the data set except for AR (Figure 5C), implying that 3D information can make up for the shortage of the training data set, thereby contributing to the higher prediction capability. Note that since we obtained the 3D data set merely by z-stacking the same H&E tissue samples, no additional cases were added to increase the training data set, thus demonstrating the cost-effectiveness of 3DHistoNet.

### Morphological Examination of Prognostic Biomarkers Using Attention Visualization

We assessed the interpretability of the model prediction by reviewing the attention maps produced together with the classification results. In the attention map consisting of the raw histology image and the corresponding heatmap, regions giving major contributions to the classification are highlighted brighter, whereas darker regions suggest fewer contributions. A certified pathologist manually reviewed the attention maps of Ki67 expression. This cell proliferation marker is known to have strong associations with cell morphology [25], thereby explaining possible correlations between characteristic phenotypes and model prediction.

**Figure 6:**
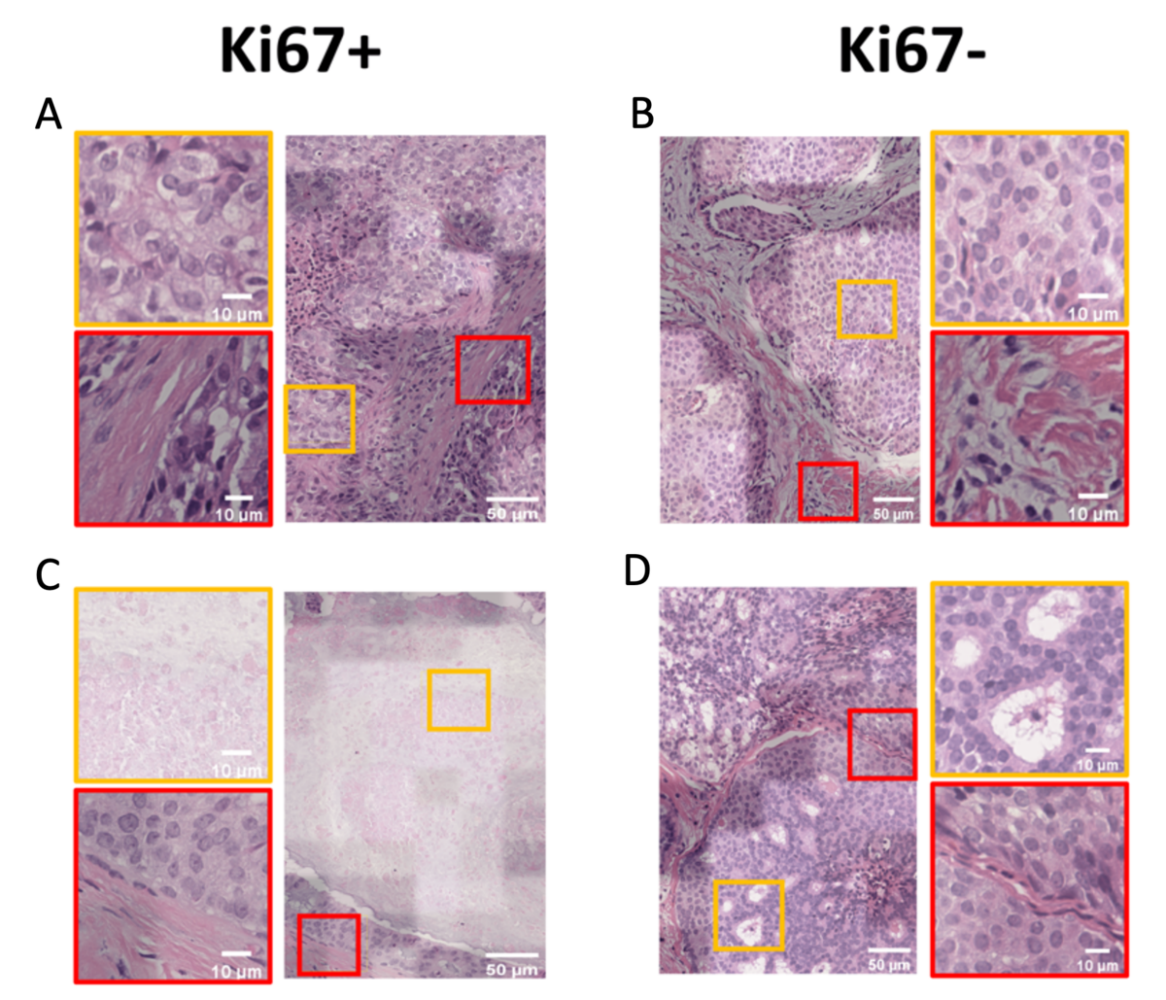
3DHistoNet can highlight discriminatory features between Ki67+ and Ki67– images by overlapping raw images with corresponding heatmaps. (A) and (B) Orange boxes show distinct cellular features for differentiation between Ki67+/– cases, which were highlighted with brighter coloration in the heatmap. The orange box in (C) highlights coagulative necrosis as a unique feature in Ki67+ cases, whereas (D) shows the lumen as a characteristic feature of the Ki67– group. Less discriminative features such as fibrosis and adipocyte were highlighted with darker coloration (red boxes in A to D).

In some Ki67+ tiles, brighter regions generally consisted of high-grade cells whose nucleoli are prominent with a large nucleus, irregular nuclear membrane, and vesicular chromatin (Figure 6A—orange box), thus indicating an active cell cycle. In contrast, the brighter regions of the Ki67– images depict smaller, round nuclei with smooth contours, suggesting cells in the dormant (G0) phase (Figure 6B—orange box). Another interesting observation is that fibrosis, a ubiquitous feature in both Ki67+/– tiles (Figure 6A, C, and D—red boxes) and adipocyte (Figure 6B—red box), which lacks characteristic morphology, are assigned lower weights (darker regions). This result suggests a high specificity of the attention map module. Other Ki67+ tiles are characterized by coagulative necrosis (Figure 6C—orange box), which occurs as a result of cell proliferation occurring faster than neovascularization, leading to localized ischemia [26]. On the other hand, Ki67– maps highlight the lumen as a unique feature (Figure 6D—orange box). Such differentiable features can also be observed in AR (Multimedia Appendix 2).

**Figure 7:**
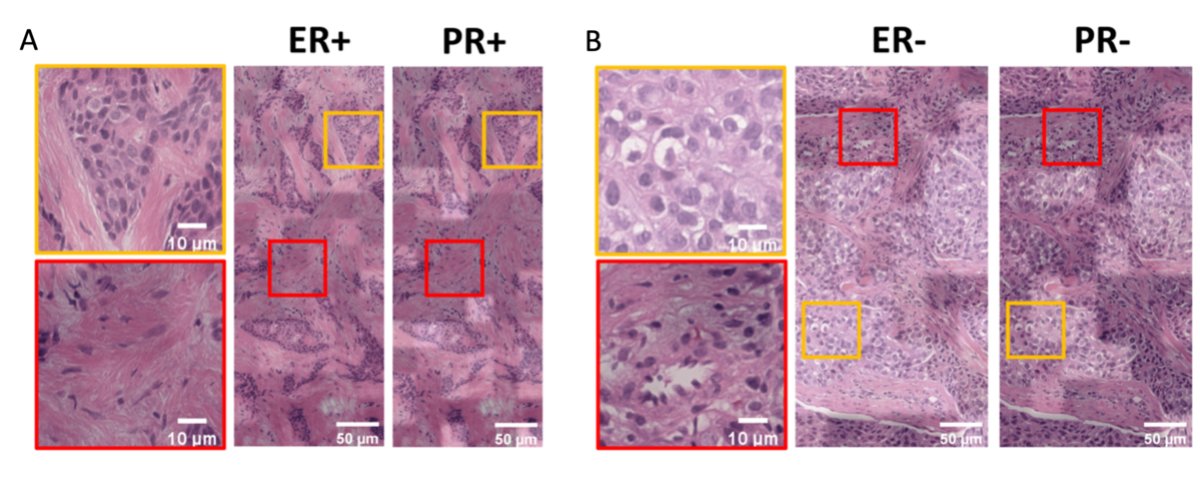
3DHistoNet can identify common features responsible for the strong positive correlation between ER and PR expressions. (A) In the ER+/PR+ image, low-grade cancer cells with ductal differentiation were commonly highlighted (orange box), whereas amorphous features of fibroblast and stroma were paid less attention (red box). (B) In the ER–/PR– image, the less growth pattern of the high-grade tumor cells was assigned brighter coloration (higher weightage, orange box), whereas cells along the blood vessels were assigned darker coloration (red box). ER: estrogen receptor; PR: progesterone receptor.

Further, given that ER expression has high positive correlations with PR [27], the attention maps of the 2 biomarkers over the same image can also identify the common features accounting for their correlation (Figure 7). In the ER+/PR+ image, the pair of attention maps assigned high weightage to the region consisting of low-grade cancer cells with lumen formation (orange box). In contrast, the stroma and fibroblast were paid less attention in both attention maps. In the case of the ER–/PR– image, high-grade cell features (enlarged nucleus, prominent nucleoli, and coarse chromatin) were highlighted, whereas cells aligned along the blood vessels were disregarded. This result supports the consistency of our attention maps with useful clinical interpretation of 3DHistoNet.

## Discussion

### Principal Findings

Breast cancer subtyping is a crucial step in determining therapeutic options, but the molecular examination based on IHC staining is expensive and time-consuming. Our data-efficient computational pathology platform, 3DHistoNet, demonstrates the capability to generate z-stacked histology images, based on which the model is trained to predict the set of all breast cancer subtypes. The main advantages of our model are that our prediction accuracy surpasses the conventional MIL model by 0.11 in terms of AUC and that such outstanding performance can be achieved with a small training data set. Finally, our platform can concurrently generate attention maps over H&E images for histopathological interpretations on the results, thereby strengthening our model’s clinical validity.

### Techniques to Improve the Generalizability of the Deep Learning Model

Training an end-to-end deep neural network to predict subtype biomarkers from z-stacked H&E scans poses challenges due to 2 factors: the large image size and the absence of pixel-level annotations corresponding to each subtype. The limited memory capacity of GPUs makes it impractical to fully use the high-resolution 3D image tiles from each specimen during training [7]. Moreover, cancer subtype prediction relies on local features that are not uniformly spread across the tissues but rather locally confined [28]. Consequently, learning features that robustly characterize cancer subtypes without manual annotations to guide pixel-level model training becomes challenging. As a result, a standard end-to-end trained deep neural network for subtype prediction is prone to suffering from memory capacity issues as well as overfitting.

To improve the memory and computational efficiency, we deployed the SSL module into our 3DHistoNet, which offers an alternative approach to extracting low-dimensional features without supervision from either specimen-level or patch-level subtype labels. Therefore, without having to feed all the patches from the whole slide image, SSL techniques can flexibly adjust the number of patches according to the given memory size of GPUs. The extracted features are shown to generalize well on all 5 prognostic biomarkers. Consequently, separate training for feature extraction of each biomarker is unnecessary, leading to higher computational efficiency.

We designed our model to be robust against the overfitting issue commonly observed in deep learning models for pathology images. First, in contrast to the standard neural network–based classification model that learns both the feature embedding and probability nodes for final end-to-end prediction, we adopted a 2-step training approach by separating the training of the embedding module from the cancer subtype prediction module, so that the number of parameters involved during each training can be reduced. Additionally, our SSL pretrained ResNet50 maps each 3D image tile from 256×256×3×17 to 1024×17, reducing the feature dimension by 192 times. Even if a sample has 50 such patches, the total number of features is only 50×1024×17, which is merely twice the number of features in a standard image with a size of 256×256×3. Therefore, despite the high dimensionality of the stacked image, the encoded features are manageable.

Further, by using a 1D convolution layer to integrate the z-stacked features, we kept the complexity of our model low, even with the large size of the image stack used. In contrast to a fully connected layer, where the number of trainable weights is directly associated with the input feature size, the 1D convolution layer allows weight sharing by traversing a small weight kernel across the feature. Therefore, when comparing the number of parameters between the models used for 2D and 3D pathology data sets, the 3D instance is only twice as large as the 2D instance. Consequently, the model complexity of the 3D instance, which impacts the risk of overfitting, is not significantly increased compared to the 2D instance.

### Validation on Performance Improvement by Histology Stack

The theoretical axial (z-axis) resolution of our whole slide scanner is about 2 μm, which is insufficient to visualize 3D tissue morphology with high resolution. Consequently, the upper or lower layers of our z-stack images show an overlap between the “in-focus” image of the upper or lower layers and the “out-of-focus” image of the middle layer, leading to an apparent blur in our image stack. Nevertheless, our results (Figures 2-5) draw a consensus that the image stack carries additional latent information that can contribute to the model performance. To verify our idea, we trained 3DHistoNet with a virtual image stack, which was prepared by blurring the “in-focus” image using an image-processing technique such that it appears to be the same as our histology image stack but empty of axial information (Multimedia Appendices 3-4). Multimedia Appendix 5 shows that our raw data set gives higher model performance compared to the virtual one, thus supporting our claim.

### Clinical Implications

The dual functions of our model to predict subtype biomarkers and generate attention maps hold several clinical values. First, the prediction of the biomarkers using H&E slides eliminates the necessity of IHC staining, saving up a substantial amount of the pathologist’s time and clinical resources. This benefit is especially valuable in the case of Ki67, whose diagnosis is time-consuming due to the manual counting of stained nuclei. On the other hand, the heatmap generated by our model identifies the characteristic features of the target biomarkers, rendering the prediction mechanism explainable and, thus, increasing the fidelity of our model to the pathologist.

### Limitations

The discriminative power of the features learned using SSL heavily depends on the choice of augmentation techniques. The augmented views from the same image should neither share too much nor too little mutual information [29]. However, finding the sweet spot is nontrivial, as it varies with both the data type [30,31] and downstream prediction task [32]. In our case, where prediction relies on cell morphology, the potential variations in morphological features across different biomarkers are unknown. If we knew such morphological discriminative features beforehand, we could further improve our model’s performance by injecting the prior knowledge during the SSL pretraining. This could be achieved either by removing augmentations that potentially “destroy” the morphological features or by adding augmentations that amplify the learning of morphological features. Hence, additional research is required to explore the optimal combination of augmentation methods that can further enhance the discriminability of the learned features.

Implementing data augmentation in our prediction module (Figure 1C) poses another challenge, as the inputs to the classifier module are low-dimensional abstract representations of image patches that we can hardly interpret. Thus, it is difficult to determine the adequate augmentations that still preserve the key discriminative features. One way to overcome the issue is to freeze the parameters in the feature extraction module (Figure 1B) and attach them to the classifier module. This way, we can perform augmentations on images and feed them directly to the classifier module; however, this would be at the cost of increased computation time and memory consumption. Therefore, it is encouraged to seek other alternatives, such as directly augmenting representations with interpolation and extrapolation [33] or turning the outputs of the feature extraction module to follow a tractable distribution with more control [34].

### Conclusion

In conclusion, we developed a data-efficient, stand-alone pathology platform, 3DHistoNet, which enables the generation of a z-stacked histology image data set and SimCLR-based prediction for 5 breast cancer subtype biomarkers.

We show that 3DHistoNet significantly outperformed the IMAGENET-pretrained supervised model in the prediction of all biomarkers, even with a limited sample size. Our model simultaneously generates attention heatmaps that are indicative of the correlation between biomarker expression and histomorphological characteristics, which would render the H&E image with higher interpretability to promote the morphology-based diagnosis among pathologists. The implementation of 3DHistoNet would encourage morphology-based diagnosis, which is faster, cheaper, and less error-prone compared to the protein quantification method based on IHC staining.

## Appendix

Multimedia Appendix 1 Mathematical definitions.

Multimedia Appendix 2 3DHistoNet can highlight discriminatory features between AR+ and AR– images. (A) In the AR+ image, abundant granular eosinophilic or vacuolated cytoplasm with distinct cell borders were highlighted as distinct features that contribute to the prediction of the image as AR+ (orange box). In contrast, regions consisting of fibroblast, stroma, and cells with high nucleus-to-cytoplasm ratio were not identified as characteristic features (red box). (B) In the AR– image, basal-like or medullary patterns with high-grade cancer cells were the key features (orange box), whereas blood vessel and condensed tumor cells were highlighted as being less prominent (red box). AR: androgen receptor.

Multimedia Appendix 3 Examples of a raw histology image stack.

Multimedia Appendix 4 Examples of a virtual image stack prepared by blurring the "in-focus" image of a raw stack to an increasing degree. Note that despite their similar appearance, the raw stack should contain more morphological information than the virtual stack.

Multimedia Appendix 5 Comparison of the 3DhistoNet performance with z-scanned and virtual image stack in terms of ROC-AUC. In general, training with the z-stacked image gives higher model performance compared to the virtual dataset.

## Abbreviations

AR: androgen receptor
AUC: area under the curve
CNN: convolutional neural network
ER: estrogen receptor
H&E: hematoxylin and eosin
HER2: erb-B2 receptor tyrosine kinase 2
IHC: immunohistochemistry
IMAGENET: ImageNet-pretrained ResNet50
MIL: multiple instance learning
PR: progesterone receptor
SimCLR: Simple Framework for Contrastive Learning of Visual Representations
SSL: self-supervised learning

## Data Availability

All code was implemented in Python with PyTorch being the primary deep learning package. Code, trained models, and scripts to reproduce the experimental result of this paper are available on the web [35]. All source code is provided under the GNU GPLv3 free software license.
