# Supplementary material for: Data-Efficient Computational Pathology Platform for Faster and Cheaper Breast Cancer Subtype Identifications: Development of a Deep Learning Model
Source: JMIR Cancer. 2023 Sep 5;9:e45547. doi: 10.2196/45547 (PMC10509735; doi:10.2196/45547)
Supplement: Multimedia Appendix 1 [file cancer_v9i1e45547_app1.docx]

In this supplementary information section, we provide further detail of our proposed 3DHistoNet with a comprehensive formulation of our model architecture and training objective for both the self-supervised feature extraction and attention-based subtype prediction.

**Methods**

**Self-supervised Feature Extraction**

We propose self-supervised learning (SSL) approach to extract feature embeddings from the H&E image stacks in a label-free way. Specifically, we adopted the recently proposed Simple framework for Contrastive Learning of visual Representations (SimCLR) [16] as our SSL framework. The overview of the SimCLR framework is shown in Fig. 1b of the main manuscript. SimCLR learns to extract latent features from H&E scans by maximizing the “agreement” between the altered views of the same image. Formally, let $X_{i}\in\mathbb{R}^{3 \times D \times pH \times pW}$be an arbitrary z-stacked tile that is sampled from an $i$th specimen with 3 RGB channels, z-stack size $D$, tile height $pH$ and width $pW$. We first randomly sample one of the D slices $x_{i}\in\mathbb{R}^{3 \times pH \times pW}$ from the tile sample $X_{i}$. Thereafter, 2 views with an identical input $t_{1}\left( x_{i} \right)$and $t_{2}\left( x_{i} \right)$are generated by a random image augmentation $t \sim T$ where $T$ is the set of all possible augmentations that are defined. The two views are then mapped to latent features $z_{i}^{\Delta}$, $z_{i}^{+ve}$ using a standard 2D CNN model $f_{\theta}: \mathbb{R}^{3 \times pH \times pW}⟼ \mathbb{R}^{C}$ parameterized by $\theta$ where $C$ is the output channel size. The objective function to train the feature extraction model $f_{\theta}$ is InfoNCE loss [17] which approximates mutual information between random variables with a tight upper bound. InfoNCE (where NCE stands for Noise-Contrastive Estimation) is defined as

$$L_{NCE}= -log\sum_{i=1}^{B} \frac{exp(sim(z_{i}^{\Delta}, z_{i}^{+ve}) )}{exp(sim(z_{i}^{\Delta}, z_{i}^{+ve}) )+ \sum_{j=1}^{B} \mathbb{I}\left[ i \neq j \right] exp(sim(z_{i}^{\Delta}, z_{j}) )}$$

1. Where $sim$ is a cosine distance metric with temperature parameter $\tau$ $(i.e. sim \left( x1, x2 \right)= \frac{<x1, x2>}{\tau\left| \left| x1 \right| \right| ||x2||}$ ), B is the mini-batch size, $\mathbb{I}$ is an indicator function and $z_{j}$ is a latent feature that includes both $z_{j}^{\Delta}$ and $z_{j}^{+ve}$. Intuitively,InfoNCE loss can be viewed as a differentiable metric that measures how accurately a model can find the true matching pair ${(z}_{i}^{+ve}, z_{i}^{\Delta})$from a sea of non-matching keys ${{\{z}_{j}\}}_{j\neq i}$ [18].

**Attention-based Prediction Model**

Given a 2D CNN model $f_{\theta}$ that is pre-trained using the SimCLR technique, the next step is to train a separate neural network $h_{\varphi}$ that uses the extracted features to predict different cancer subtypes (Figure 1C of the main manuscript). To reduce memory consumption and shorten computation time during training, instead of composing the classifier network with the “frozen” $f_{\theta}$, we first map 3D H&E tiles to embedding $\left\{ Z_{n}\in\mathbb{R}^{D \times C} \right\}_{n=1}^{M}$ where $M$ is the number of embedded tiles for a specimen.

Prediction model $h_{\varphi}$, at a higher level, is a composition of 3 sub-modules: 1) 1D CNN that integrates $D$ slice embeddings into a single embedding, 2) attention module that generates a heatmap which indicates the relative importance across the embedded tiles, and 3) a classifier layer that generates probability outcome.

The embeddings of a 3D H&E image $\left\{ Z_{n} \right\}_{n=1}^{M}$ can be regarded as a set of multi-channel 1D signals that potentially contain informative interactions across the signal. 1D CNN module is a good model choice for discovering such interactions. Our 1D CNN module is a composition of two 1D CNN blocks. Each block contains 1D convolutional layer with kernel size 3, followed by a Rectified Linear Unit layer. The 1DCNN module produces pooled embeddings $\left\{ \bar{z}_{n}\in\mathbb{R}^{C} \right\}_{n=1}^{M}$. In the attention module, we measure the relative importance across the tile embeddings $a\in\left[ 0,1 \right]^{M}$ and use the importance to perform a weighted average across the embeddings to produce$h$ = $\sum_{n=1}^{M} a_{n}*\bar{z}_{n}$. The attention score $a_{n}$ of the n-th tile is defined as [19].

$$a_{n}= \frac{exp\{ W \left( \tanh\left( V \bar{z}_{n}^{T} \right) \odot\text{sigm}\left( U \bar{z}_{n}^{T} \right) \right)\}}{\sum_{n=1}^{M} exp\{ W \left( \tanh\left( V \bar{z}_{n}^{T} \right) \odot\text{sigm}\left( U \bar{z}_{n}^{T} \right) \right)\}}$$

Where $W, U,V\in\mathbb{R}^{C}$ are trainable weights. Finally, in the classifier module, the attention pooled embedding $h$ is fed into a fully connected layer followed by a softmax operation to produce a subtype probability. All the 3 sub-modules are trained end-to-end using cross-entropy loss with the ground truth cancer subtype labels (ER, PR, AR, HER2 and Ki67).
