# Supplementary figures and images for: Data-Efficient Computational Pathology Platform for Faster and Cheaper Breast Cancer Subtype Identifications: Development of a Deep Learning Model
Source: JMIR Cancer. 2023 Sep 5;9:e45547. doi: 10.2196/45547 (PMC10509735; doi:10.2196/45547)

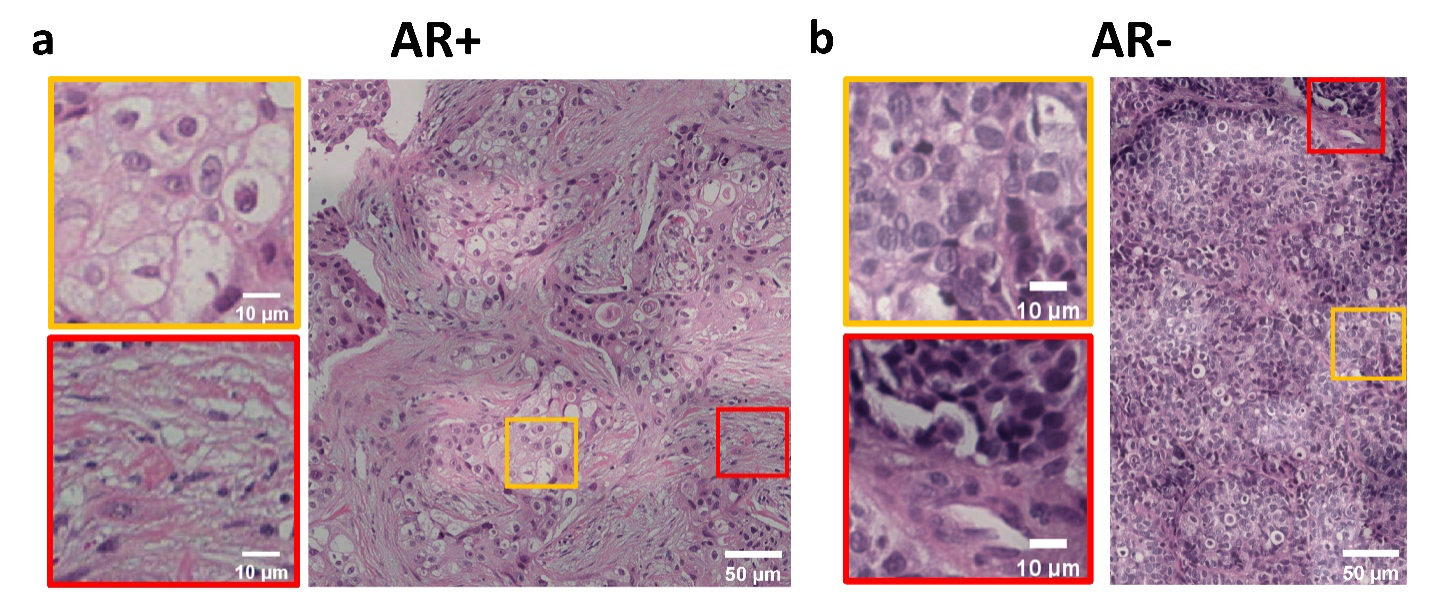

Supplement: Multimedia Appendix 2 [file cancer_v9i1e45547_app2.png]

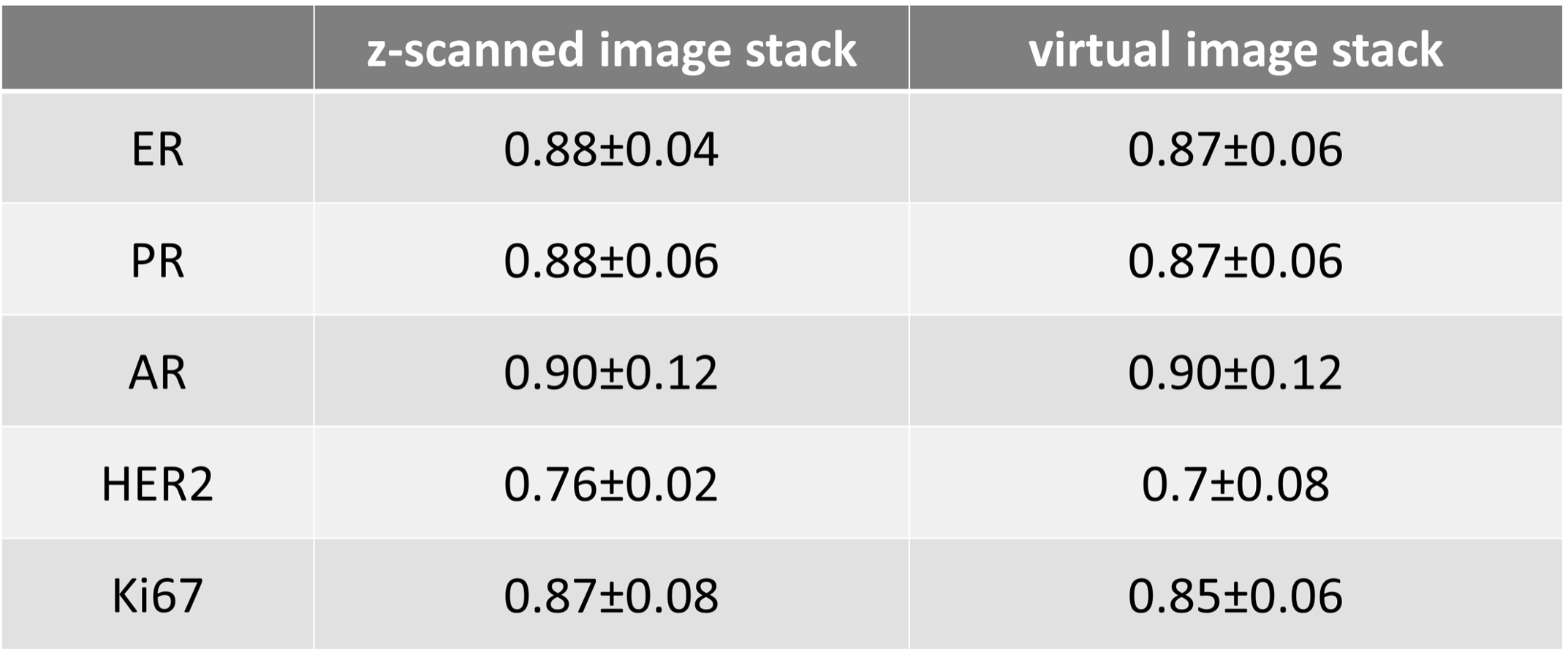

Supplement: Multimedia Appendix 5 [file cancer_v9i1e45547_app5.png]
